# GOx/Hb Cascade Oxidized Crosslinking of Silk Fibroin for Tissue-Responsive Wound Repair

**DOI:** 10.3390/gels8010056

**Published:** 2022-01-12

**Authors:** Hongdou Shen, Pei Wang, Xiaoke Han, Mengli Ma, Yinghui Shang, Ye Ju, Saiji Shen, Feng Yin, Qigang Wang

**Affiliations:** 1 Department of Joint Surgery, Shanghai East Hospital, School of Medicine, Tongji University, Shanghai 200120, China; shenhongdou2018@163.com (H.S.); drwp007@163.com (P.W.); 2 School of Chemical Science and Engineering, Tongji University, Shanghai 200092, China; 1931002@tongji.edu.cn (X.H.); 17334567254@163.com (M.M.); shangdiyinghui@163.com (Y.S.); juye1997@hotmail.com (Y.J.); shensaiji@tongji.edu.cn (S.S.)

**Keywords:** silk fibroin, antioxidative hydrogels, cascade oxidized crosslinking, tissue-responsive, wound regeneration

## Abstract

Promising wound dressings can achieve rapid soft-tissue filling while refactoring the biochemical and biophysical microenvironment to recruit endogenous cells, facilitating tissue healing, integration, and regeneration. In this study, a tissue biomolecule-responsive hydrogel matrix, employing natural silk fibroin (SF) as a functional biopolymer and haemoglobin (Hb) as a peroxidase-like biocatalyst, was fabricated through cascade enzymatic crosslinking. The hydrogels possessed mechanical tunability and displayed adjustable gelation times. A tyrosine unit on SF stabilised the structure of Hb during the cascade oxidation process; thus, the immobilized Hb in SF hydrogels exhibited higher biocatalytic efficiency than the free enzyme system, which provided a continuously antioxidative system. The regulation of the dual enzyme ratio endowed the hydrogels with favourable biocompatibility, biodegradability, and adhesion strength. These multifunctional hydrogels provided a three-dimensional porous extracellular matrix-like microenvironment for promoting cell adhesion and proliferation. A rat model with a full-thickness skin defect revealed accelerated wound regeneration via collagen deposition, re-epithelialisation and revascularisation. Enzyme-loaded hydrogels are an attractive and high-safety biofilling material with the potential for wound healing, tissue regeneration, and haemostasis.

## 1. Introduction

Skin wounds can transform into chronic or non-healing wounds without undergoing orderly and timely repair to recover structural and functional integrity; this induces pain and results in an economic and clinical burden [1,2,3]. Wound regeneration following injury is a complicated and metabolically demanding process, which progresses through four phases: haemostasis, inflammation, proliferation, and tissue remodelling [4,5,6]. Filling materials designed for tissue regeneration should be loaded with biochemical and biophysical factors to modulate the extracellular microenvironment and promote the spreading, proliferation, and differentiation of endogenous cells [7]. Hydrogels are attractive wound dressings because of their excellent moisture retention, controllable mechanical properties, and favourable biocompatibility, and can be used as biologically active substances to better simulate the skin extracellular matrix [8,9,10,11]. In particular, injectable hydrogels possess in situ formability for filling complex tissue defects, providing local biological and mechanical signals to enhance tissue regeneration, which can be easily manipulated in vivo by minimally invasive surgery to reduce suffering [12,13]. Natural proteins and polysaccharides with excellent biocompatibility, such as gelatin [14], collagen [15], hyaluronic acid [16], and chitosan [17], are preferred candidates for injectable hydrogels. The physical, chemical, and biochemical reactions triggered by environmental factors, including pH [18], temperature [19], light [20], and enzyme [21,22,23] have been used to generate injectable hydrogels. However, it is difficult to achieve multifunctional synergy such as rapid gelation, adjustable mechanical properties, anti-inflammatory properties, and interaction with tissues with currently available materials, which restricts their use in clinical applications. There is an urgent need for an efficacious strategy to fabricate bioactive hydrogels to satisfy all stages of the healing process and undergo sol–gel transition under physiological conditions. Thus, it is an attractive idea to fabricate materials by utilising biomolecules and biochemical reactions within the wound, subsequently regulating inflammation and tissue regeneration.

In organisms, reactions catalysed by enzymes under normal physiological conditions can help to maintain the biological activity of biomolecules. Enzymatic in situ crosslinking hydrogels possess great biocompatibility and represent an emerging approach for tissue engineering; moreover, the reaction can be directly regulated by modulating enzyme activity [24,25,26,27]. Free haemoglobin, a component of blood, possesses peroxidase activity accompanying the generation of protein radicals during the peroxidative consumption of hydrogen peroxide (H_2_O_2_). The globin crosslinking of Hb can stabilise the radical burden to form an irreversible complex, resulting in potential anti-oxidative activity; thus, it functions as an antioxidative peroxidase during peroxidative stress [28]. Silk fibroin (SF) is a tyrosine-rich fibrous protein found abundantly in natural silkworm cocoons with favourable mechanical properties, biocompatibility, biodegradation, and minimal inflammation, which can be used as a cell support matrix and scaffold for the regeneration of bone, cartilage, skin, nerves, and blood vessels [29]. From the perspective of communication with the tissue, the enzyme-crosslinked SF hydrogels may enable the rapid filling of the wound area to refactor the biochemical and biophysical microenvironment of wounded tissue.

**Figure 1:**
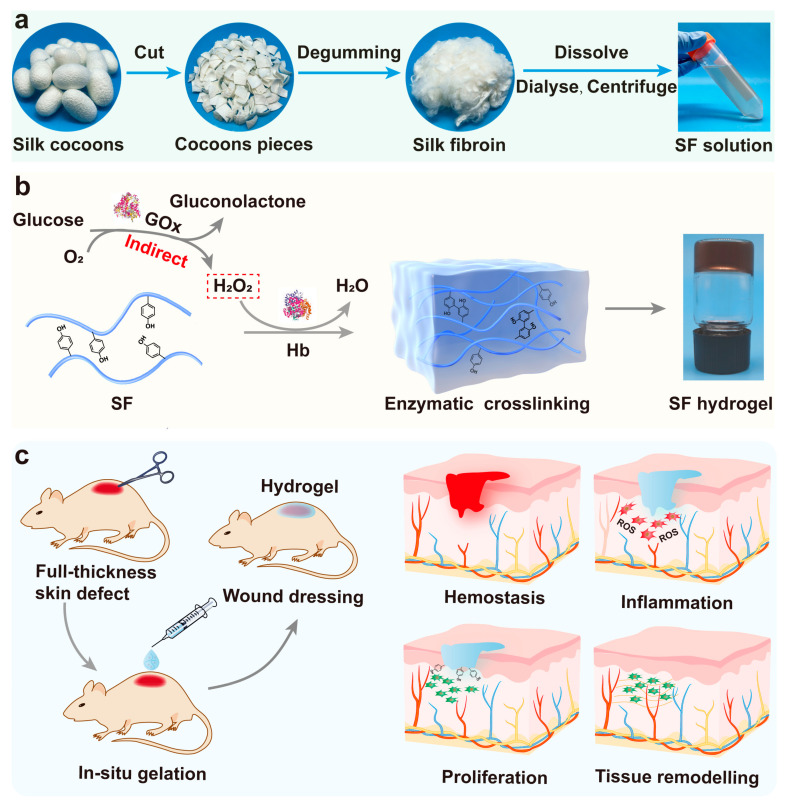
Schematic diagram illustrating silk fibroin (SF) hydrogel fabricated by enzymatic crosslinking for wound healing. (**a**) The extraction of SF solution by degumming, dissolving, and dialyzing from Bombyx mori cocoons. (**b**) Reaction mechanism of Hb-mediated covalent crosslinking between tyrosine residues on silk. (**c**) Application of SF hydrogels as wound dressings for skin wound regeneration.

Herein, inspired by the process of antioxidation in living organisms, we have proposed a new in situ cascade enzymatic crosslinking method to transform regenerated SF solution into hydrogels for wound healing by utilizing endogenous components within the organism. (Figure 1). In the SF/glucose/glucose oxidase (GOx)/Hb system, GOx catalyzed the conversion of O_2_ and biomolecular glucose into H_2_O_2_ and gluconolactone. Then, as a peroxidase-like biocatalyst in the presence of H_2_O_2_, Hb initiated an enzymatic cascade to fabricate SF hydrogels through the formation of dityrosine bonds. These hydrogels exhibited mechanical tunability, rapid gelation behaviour, high biocompatibility, and biodegradability. Furthermore, the mechanism of gelation and the pathways involved in the enzymatic free radical transfer was demonstrated. The tyrosine-rich moieties of SF stabilized the Hb structure to weaken the cytotoxicity of the trigger system, conferring to the hydrogel system continuous antioxidative activity, which promoted cell adhesion and proliferation. A rat model with a full-thickness skin defect was used to evaluate the feasibility of these multifunctional SF hydrogels as wound dressings.

## 2. Results and Discussion

### 2.1. Fabrication and Structural Characterisation of Hb-Mediated SF Gelation

The SF solution was extracted by degumming, dissolving, and dialyzing from natural silk (Figure 1a). In the presence of H_2_O_2_, ferric Hb (metHb) exhibits peroxidase-like enzymic activity; Hb (Fe^III^) is oxidised to higher oxidation states Hb (Fe^IV^), which is accompanied by the generation of protein radicals and H_2_O [30]. It has been suggested that the protein radicals are tyrosyl radicals, resulting in the formation of protein dimers as key intermediates [31]. H_2_O_2_ was produced in situ by continuous enzymatic generation using GOx. Peroxide-induced protein radicals on Hb can capture a hydrogen atom from the tyrosine on the SF side chains to generate tyrosyl radicals, and then form dityrosine bonds by tyrosine radicals reacting with one another (Figure 1b). For example, 1SF-10G represents the hydrogel prepared using 1% *w*/*v* SF solution as a biopolymer, 4.5 mg mL^−1^ glucose, 10 mu mL^−1^ GOx, and 3 mg mL^−1^ Hb as the initiator (Table S1). The vial inversion test demonstrated that increasing both the SF and GOx concentrations resulted in adjustable gelation times from ~5–30 min (Figure S1).

**Figure 2:**
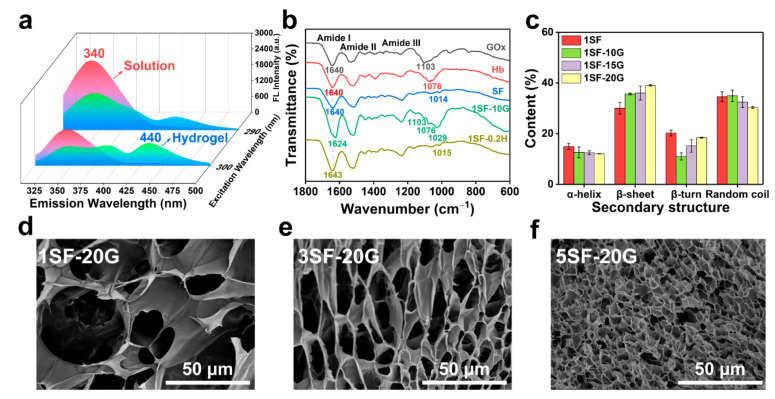
Mechanism of SF hydrogel formation and characterization of hydrogel morphology. (**a**) Fluorescence excitation-emission spectra of SF solution and SF/glucose/GOx/Hb hydrogel. (**b**) FTIR spectra of GOx, Hb, SF, and 1SF-10G. (**c**) Content of second structure of SF and SF/glucose/GOx/Hb hydrogels by peak-fitting. The 3D porous structure of SF hydrogel: (**d**) 1SF-20G, (**e**) 3SF-20G, (**f**) 5SF-20G. Error bars represent the mean ± standard deviation (s.d.); *n* ≥ 3.

The dityrosine bonds were assessed by fluorescence spectroscopy and exhibited a maximum peak at an emission wavelength of 340 nm when excited at 290 nm. A new maximum peak appeared in the absorption spectrum at an emission wavelength of 440 nm when excited at 300 nm after gelation, which indicated the reduction of tyrosine and the formation of dityrosine bonds (Figure 2a) [32]. The FTIR spectrum displayed the characteristic absorption peaks of amide I, II, and III bands (Figure 2b and Figure S2a). There was a peak shift of the amide I band from 1640 cm^−^^1^ to 1624 cm^−^^1^ in the SF hydrogels, suggesting conformational changes in the silk secondary structure from a random coil to crystalline β-sheets [33]. The resultant secondary structures of the SF hydrogels by peak-fitting (Figure S2b, 1600−1700 cm^−^^1^) suggested an increased content of β-sheets compared with pure SF protein (Figure 2c and Figure S2c,d) supporting the formation of hydrogels. To confirm the morphology and structure of the hydrogels, scanning electron microscopy (SEM) images revealed a 3D porous network of SF hydrogels. The average diameters of 1, 3, and 5% SF hydrogels were 15–50, 5–15, and 3–5 μm, respectively, which could be modulated by varying the concentrations of SF and GOx to adjust the crosslinking density of tyrosine residues (Figure 2d–f and Figure S3).

### 2.2. Rheological and Mechanical Properties of SF Hydrogels

**Figure 3:**
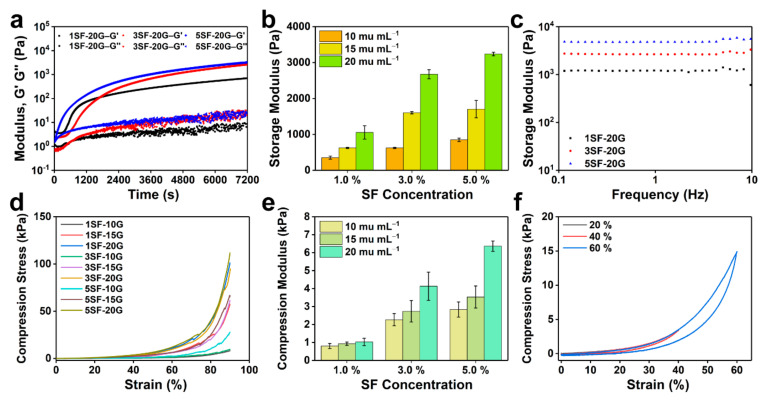
Rheological and mechanical properties of SF hydrogels. (**a**) The storage modulus (G′) and loss modulus (G″) of SF/glucose/GOx/Hb (GOx: 20 mu mL^−1^) hydrogels with different SF content under time sweep. (**b**) G′ of SF hydrogels with varying SF and GOx concentrations. (**c**) G′ of SF/glucose/GOx (20 mu mL^−1^) with different SF concentrations under frequency sweep. (**d**) The compression stress–strain curves of SF hydrogels. (**e**) The compression modulus of SF hydrogels with varying SF and GOx concentrations. (**f**) The cyclic compression stress–strain curves of the 5SF-20G hydrogels. Error bars represent the mean ± s.d.; *n* ≥ 3.

Stiffness has a significant impact on cell migration and the maintenance of cell morphology, especially in regenerative medicine and tissue engineering [34]. Figure 3 shows the gelation kinetics, shear properties, and compression performance of the SF hydrogels, which were assessed by rheology and compression tests. The storage modulus (G′) and loss modulus (G″) of the SF/glucose/GOx/Hb system under a time sweep are shown in Figure 3a and Figure S4. The storage modulus displayed time-dependent increasing within 7200 s. This indicated that GOx-triggered gelation was a continuous, moderate oxidation process. By increasing the SF concentration from 1 to 5% *w*/*v*, and the GOx concentration from 10 to 20 mu mL^−1^, the storage modulus of the hydrogels increased from 352 ± 43 to 3238 ± 50 Pa (Figure 3b). G′ was independent of the frequency sweep from 10 to 0.1 Hz as a result of the chemical crosslinking of SF, indicating that the hydrogels presented great mechanical stability (Figure 3c). Subsequently, compression stress–strain curves of the SF hydrogels were obtained to evaluate the compressive modulus (Figure 3d). As observed in Figure 3e, the compressive modulus of GOx-triggered hydrogels varied from 0.8 ± 0.14 to 6.4 ± 0.29 kPa. The 5% SF hydrogels recovered from the compressive strains of 60% with minimal hysteresis, as proven by the cyclic compression stress–strain curves (Figure 3f). These analyses suggest that SF hydrogels exhibit tunable mechanical properties and recovery by controlling the concentrations of SF and GOx which are able to simulate some of the properties of natural skin tissues.

### 2.3. Mechanism of Enzymatic Crosslinking and Antioxidant 

**Figure 4:**
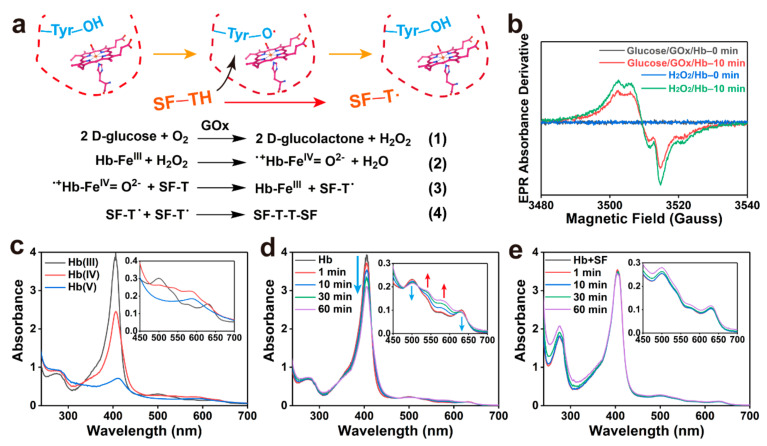
Mechanism of Hb-mediated crosslinking and SF as a protective antioxidant. (**a**) Mechanism of free radical transfer during gelation. (**b**) The EPR measurements confirmed the presence of tyrosyl radical. (**c**) Different states of Hb oxidation in the presence of H_2_O_2_ within 12 h. (**d**) The states of Hb oxidation without the addition of SF in 1 h (glucose: 0.75 mg mL^−1^, GOx: 3.33 mu mL^−1^, Hb: 0.5 mg mL^−1^). (**e**) The states of Hb oxidation with the addition of SF (1 mg mL^−1^) in 1 h.

Hb-mediated radical crosslinking was confirmed by low-temperature electron paramagnetic resonance (EPR) spectroscopy and UV−vis spectroscopy. Figure 4a depicts the free radical transfer pathways of the SF/glucose/GOx/Hb system. GOx catalysed glucose to generate H_2_O_2_ (reaction 1). When H_2_O_2_ was consumed, the metHb was oxidated to ^·+^Hb-Fe^IV^ = O^2−^ with the generation of H_2_O (reaction 2). Signals of protein radicals can be detected by EPR spectroscopy, which were confirmed to be tyrosyl radicals (Figure 4b) [35]. Radicals can abstract hydrogen atoms from the tyrosine of SF, thereby initiating a new tyrosyl radical (reaction 3). Finally, the tyrosine radicals react to form a covalent bond (reaction 4). UV−vis spectroscopy revealed the time-dependent oxidation of metHb in the presence of H_2_O_2_. Figure 4c shows the intermediate products of Hb heme group oxidation in 12 h. The original metHb presented an intense Soret band at 405 nm and a Q band at 499 and 630 nm with shoulders at 536 and 574 nm, respectively. The Hb(IV) compound presented absorption at 538 and 580 nm, and the Hb(V) compound had maximum absorption at 588 nm with a Soret band redshift [36]. Hb oxidation in the presence of GOx-generated H_2_O_2_ revealed time-dependent oxidation (Figure 4d). Furthermore, as shown in Figure 4e, the addition of SF effectively prevented the gradual oxidation of Hb, corresponding to reaction (3), SF played a significant role in reducing the amount of oxidised Hb within 1 h. Consequently, this can be depicted as a redox reaction, the existence of SF donated electrons in the processes of Hb peroxidation or drive reductive steps in pathways, which protects the Hb structure due to the abundant tyrosine residues on protein chains.

**Figure 5:**
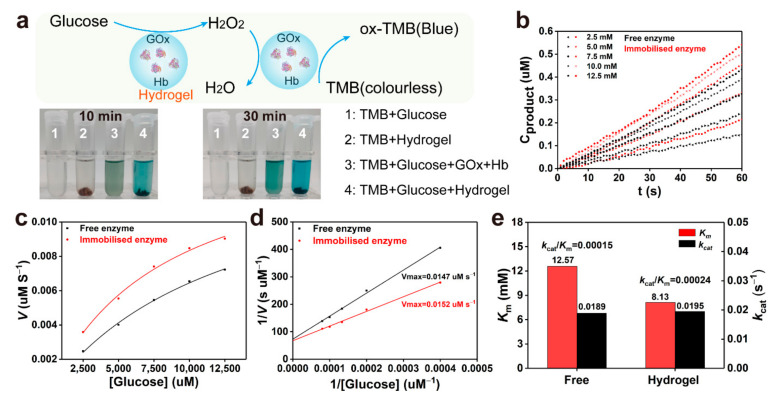
The catalytic activity of the free and immobilised enzyme. (**a**) Mechanism of tetramethylbenzidine (TMB) oxidation and time-dependent absorbance changes at 25 °C. (**b**) Catalytic reactions during the first minute of the free and immobilised enzyme with different glucose concentrations. (**c**) Michaelis–Menten kinetics of TMB oxidation catalysed by free and immobilised enzyme. (**d**) The Lineweaver–Burk plots of free and immobilised enzyme. (**e**) Comparison of catalytic activity between free and immobilised enzyme.

Furthermore, the catalytic activity of Hb in hydrogels was evaluated by the addition of glucose and using tetramethylbenzidine (TMB) as a chromogenic substrate (Figure 5a) [37]. Figure 5b compares the initial reaction rate of the free and immobilised Hb as mimetic peroxidase under different glucose concentrations, which showed that the reaction rate of the immobilised enzyme was significantly higher than that of the free enzyme, which can also be supported by the time-dependent absorbance changes. Figure 5c observed that the oxidation reaction catalysed by the free and immobilised enzyme follows a Michaelis–Menten behaviour. By using the Lineweaver–Burk plots, the *K*_m_ value of the immobilised enzyme in the hydrogels was smaller than that of the free enzyme indicating a higher affinity of the substrates for the hydrogels (Figure 5d) [38]. The *k*_cat_/*K*_m_ value of the immobilised enzyme showed 1.6-fold catalytic effiency of the free enzyme system (Figure 5e), which suggested the Hb retained high biocatalytic activity in the hydrogels. These enzyme-loaded SF hydrogels demonstrate a promising antioxidative function during peroxidative stress.

The oxidative microenvironment and inflammation within the wound generate more reactive oxygen species (ROS), which hinder the recovery of wound tissue [39]. To overcome this, the antioxidative activity of the SF hydrogels (5SF-10G and 5SF-20G) in vitro were confirmed. First, the scavenging rate of multiple ROS including H_2_O_2_, DPPH, and hydroxyl radicals (·OH) of the SF hydrogels (Figure S5) were evaluated and the results showed that the scavenging rate of ·OH, DPPH and H_2_O_2_ was 74.7–80.2, 50.7–53.1, and 44.3–48.6% (Figure S6), respectively, which indicated that the SF hydrogels possessed a high antioxidative capacity through regulating the enzyme concentration. The results suggested that SF allows the Hb/SF complex to function as a protective sink within ROS-exposed tissues. The temporal and spatial control of biodegradation is important for tissue regeneration [40]. In vitro degradation experiments were performed using pronase E and 5% SF hydrogels retained ~70% their original weight at day 7 (Figure S7).

### 2.4. Cell Viability of Enzymatic System and Hydrogels

**Figure 6:**
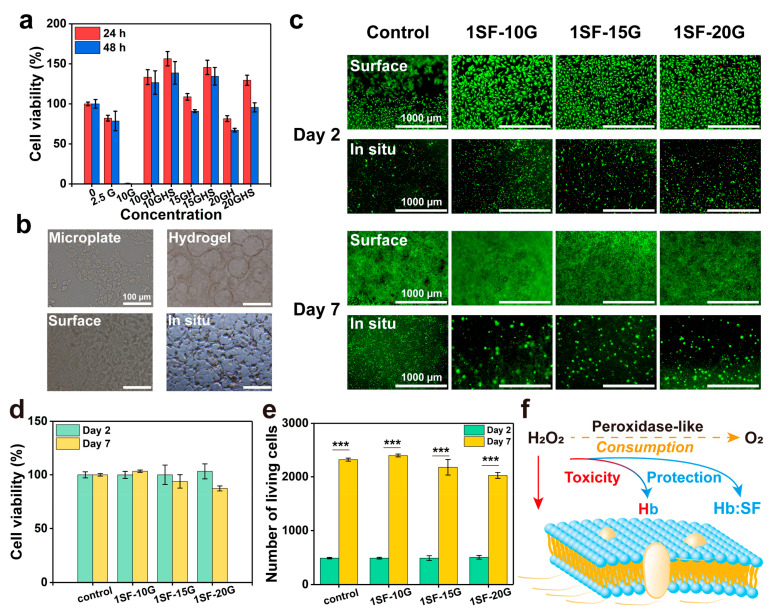
Survival and proliferation of NIH 3T3 cells. (**a**) Cell viability of the SF/glucose/GOx/Hb system in 24 and 48 h. (2.5G: GOx [2.5 mu mL^−1^], 10G: GOx [10 mu mL^−1^], 10GH: GOx/Hb [10 mu mL^−1^, 3 mg mL^−1^], 10GHS: GOx/Hb/SF [10 mu mL^−1^, 3 mg mL^−1^, 1 mg mL^−1^]). (**b**) Coloured bright field of cells and hydrogel. Scale bars are 100 µm. (**c**) Cell proliferation of NIH 3T3 cultured on the hydrogel surface and encapsulated in SF hydrogels, live (green) and dead (red) cell staining on hydrogels over a 7-day period, scale bars are 1000 µm. (**d**) Cell viability and (**e**) number of living cells cultured on the surface of SF hydrogels (days 2 and 7). (**f**) Schematic diagram of cell protection mechanism during the gelation. *** *p* < 0.001.

The highly hydrated tissue-like microenvironment and 3D porous structure of the hydrogel modulate cell spreading and adhesion, which regulate cell survival and proliferation to ensure tissue regeneration [41]. First, the enzymatic initiation system and reactant precursor should possess the desired cytocompatibility. A previous study investigated the H_2_O_2_-rich microenvironment that can induce cell damage under various pathophysiological conditions, while the addition of Hb was regarded as a complicated biological reaction, and whether it is cytotoxic is dependent on the balance of H_2_O_2_ consumption and the generation of protein radicals [42]. Therefore, we initially estimated cellular metabolism after treatment of the enzymatic system for 24 and 48 h using the cell counting kit-8 (CCK-8) assay. Figure 6a presents the cell viability of the GOx-triggered systems. Cells were not viable following incubation with GOx (10 mu mL^−1^) in the culture medium containing 4.5 mg mL^−1^ glucose for 24 h. The addition of Hb (3 mg mL^−1^) was able to overcome the toxicity of GOx (10 mu mL^−1^) due to the consumption of H_2_O_2_ by the redox reaction of heme and the successful scavenging of protein radicals by globin crosslinking [28]. The decreased cell viability revealed the restricted protective ability of Hb with increasing GOx concentrations (15, 20 mu mL^−1^). Hence, the presence of SF (1 mg mL^−1^) maintained cell viability at 95% with a high GOx concentration (20 mu mL^−1^) for 48 h, indicating that SF attenuated excessive cell damaged triggered by Hb peroxidation. Briefly, synergy between Hb and SF gradually reduced the cytotoxicity of H_2_O_2_ to provide a highly biocompatible trigger system.

Cell adhesion, survival, and proliferation were assessed using live/dead staining. The 1% SF (the lowest concentration that can form hydrogel) was chosen to study whether a low concentration of SF can withstand the cytotoxicity caused by GOx-generated H_2_O_2_ and excessive Hb protein radicals during the gelation process. As shown in Figure 6b, compared with the cells on the microplate, the cells spread on the hydrogel surface and exhibited elongated or branched morphologies, indicating favourable interactions between the cells and the hydrogel matrix. The cells were spread on the hydrogel surface and encapsulated in hydrogels with increasing GOx concentrations to evaluate viability on day 2 and after 1 week (Figure 6c). Cells seeded onto hydrogels showed more uniform adhesion than those on the control microplate surface. Fewer dead cells and a larger spreading area were observed on the surface at lower GOx (10 mu mL^−1^) concentrations. Compared with the control microplate group, more than 100% of the cells survived on day 2, and 90% of the cells still lived on the surface of GOx-triggered hydrogels on day 7 (Figure 6d). Cells on hydrogels exhibited an approximately 4.8-fold proliferation over a 7-day period (Figure 6e). In addition, for in situ encapsulation, the metabolic activity of GOx-triggered hydrogels was higher than that of the control group (SF solution) on day 2 (Figure 6c); moreover, larger cell clusters were observed in the hydrogel groups on day 7. The mechanisms underlying the toxicity and protection of H_2_O_2_, Hb, and the Hb-SF complex are shown in Figure 6f. The addition of Hb and Hb-SF complex progressively reduced the toxicity of H_2_O_2_ by Hb crosslinking and shielded excessive Hb protein radicals by SF crosslinking. This provided protection against toxicity induced via two pathways. The SF/glucose/GOx/Hb system offered a moderate method to achieve the balance of H_2_O_2_ consumption and generation, which avoided more cell damage and enzyme inactivation immediately exposed to excessive levels of H_2_O_2_ compared to the previously H_2_O_2_-triggered crosslinked system [25]. Furthermore, we investigated the intracellular ROS levels in the oxidative microenvironment to demonstrate the protective function of SF hydrogels against ROS. Low fluorescence intensity was observed in NIH 3T3 cells treated with hydrogels, and the fluorescence intensity was similar to that of the control group, indicating that the hydrogels exhibited a high ROS scavenging rate (Figure S8).

### 2.5. Enzymatically Crosslinked SF Hydrogels for Full-Thickness Wound Regeneration

**Figure 7:**
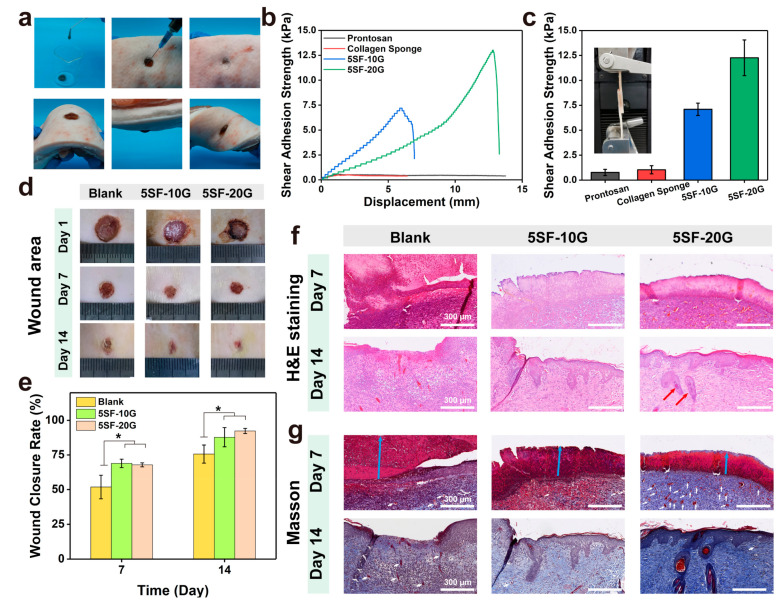
Wound healing ability of SF hydrogels. (**a**) The injectability and formability of SF hydrogels. (**b**) The adhesion strength curves of liquid wound dressing (Prontosan), collagen sponge, 5SF-10G, and 5SF-20G to porcine skin. (**c**) Adhesion strengths of Prontosan, collagen sponge, 5SF-10G, and 5SF-20G to porcine skin, *n* = 3. (**d**) Photograph of wound area treated by hydrogels on days 1, 7, and 14. (**e**) Rate of wound healing following treatment by hydrogels on days 7 and 14. (**f**) Haematoxylin and eosin (H&E) staining of the wound tissues on days 7 and 14. (**g**) Masson’s trichrome (Masson) staining of the wound tissues on days 7 and 14, blue and white arrows represent the residual scab and vessels, respectively. Scale bars represent 300 μm. Error bars represent the mean ± s.d.; *n* = 3, * *p* < 0.05.

Dressings with desirable self-standing capability and tissue adhesion strength can easily cover the wound tissues to provide a beneficial microenvironment for tissue healing. As shown in Figure 7a, Movie S1 and S2, the SF hydrogels had great injectability and formability to support the wound tissues and bear various deformation. In addition, the shear adhesive strengths of the SF hydrogels to porcine skin were evaluated via the lap-shear testing. The 5SF-10G and 5SF-20G hydrogels showed the adhesive strength of 7.1, and 12.3 kPa, respectively, which were higher than that of commercial Prontosan wound gel and collagen sponge (0.8, and 1.0 kPa, respectively). The results demonstrated that the SF hydrogels possess promising self-standing capability and tissue-adhesive strength, which can completely cover and support the wound tissue to prevent secondary infection.

Because the hydrogels possessed the anticipated multifunctional properties required for a wound dressing, the wound healing ability of SF hydrogels was evaluated using a full-thickness skin defect model. Considering the gelation time, mechanical stability, and adhesion strengths, two types of hydrogels (5SF-10G and 5SF-20G) were chosen as the experimental groups. The wounds (8 × 8 mm) were covered by in situ gelation. First, the rate of relative wound closure was observed using photographs (Figure 7d). The rate of closure (Figure 7e) in the untreated blank group was 51.8% on day 7, which was significantly lower than that of the two experimental groups (68.9, 67.8%, respectively). Moreover, the blank group still retained obvious wounded areas on day 14, with a closure rate of 75.6%. Conversely, the closure rate of hydrogel groups could reach 87.8% and 92.3%, which indicated that the hydrogels, especially for the 5SF-20G hydrogels, promoted wound closure and shortened the time to wound healing. To evaluate the extent of wound regeneration, hematoxylin and eosin (H&E) and Masson’s trichrome (Masson) staining were performed on skin tissues collected on days 7 and 14. As observed in Figure 7f, inflammatory cells were observed in all groups; however, more inflammatory cells infiltrated the wound tissue in the blank group compared with that in the hydrogel groups on day 7. This was due to the antioxidative performance of SF hydrogels, which was beneficial to diminish inflammatory responses. Granulation tissue (green double-headed arrows) formed at the wound tissues was also significantly enhanced in the 5SF-20G hydrogel group on day 7, which was thicker than that of the blank group and 5SF-10G hydrogel group (Figure S9a). On day 14, inflammatory cells and discontinuous epidermal tissues still existed in the blank groups, suggesting the wound was still in the proliferation phase of repair; in contrast, apparent continuous epidermal tissue was observed in all hydrogel groups and the 5SF-20G group presented more mature dermal tissue with the appearance of hair follicles (red arrows), which indicated the wound had entered the remodelling stage. Masson staining was used to visualise collagen in the proliferative connective tissue. The blank and 5SF-20G groups exhibited a loose collagen arrangement and bleeding on day 7, while the 5SF-20G group showed the deepest blue colour of stained collagen among all groups. After 14 days of treatment, the collagen arrangement in the blank group remained irregular and loose, while higher collagen deposition was observed in the hydrogel groups. Obviously, the 5SF-20G group exhibited denser collagen deposition compared to the 5SF-10G group (Figure 7g and Figure S9b). Residual scabs were still observed in all groups; the group treated by 5SF-20G had thinner scabs (blue arrows) than the other groups, these results were consistent with the residual area of the wound observed in the photograph. All results indicated that GOx-triggered hydrogels, especially for 5SF-20G, possessed greater healing efficiency than the other hydrogels, which was attributed to the rapid gelation, mechanical stability, high adhesion strength, and ROS regulation that promoted epidermal–dermal tissue regeneration and collagen deposition, which facilitated wound healing.

**Figure 8:**
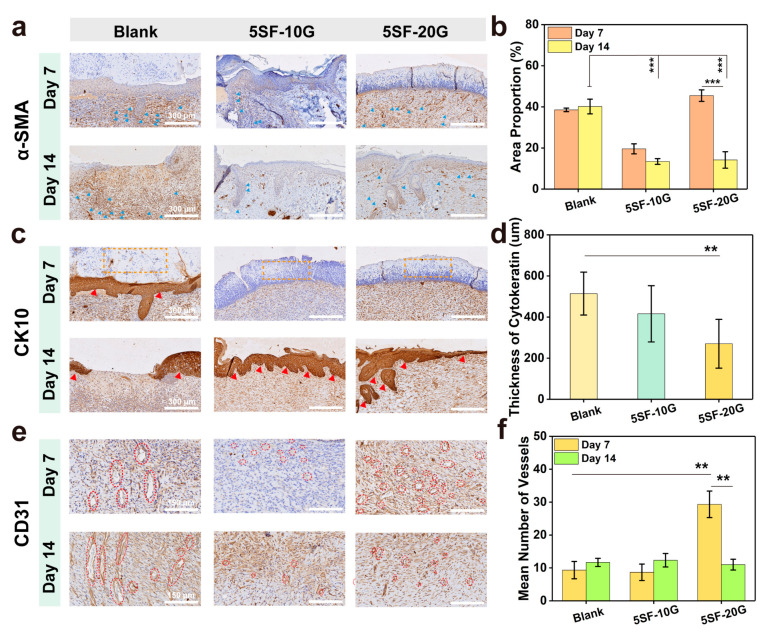
Immunohistochemical staining of wounds treated with hydrogels. (**a**) α-smooth muscle actin (α-SMA) staining, showing myofibroblasts on days 7 and 14, blue triangles indicated the positive expression of α-SMA, scale bars are 300 μm. (**b**) CK10 staining, showing keratin in skin on days 7 and 14, orange dotted frames and red triangles indicated the negative and positive expression of keratin, respectively, scale bars are 300 μm. (**c**) CD31 staining images, representing the extent of vascularisation on days 7 and 14, red dashed circles indicated the vessels, scale bars are 150 μm. (**d**) The relative density of α-SMA on days 7 and 14. (**e**) The cytokeratin thickness (μm) of wounded tissue on day 14. (**f**) Mean number of vessels calculated from CD31 staining. Error bars represent the mean ± s.d.; *n* = 3, ** *p* < 0.01, *** *p* < 0.001.

The cellular and molecular mechanisms underlying wound repair was also examined by immunohistochemical (IHC) staining. α-smooth muscle actin (α-SMA) is a reliable marker of myofibroblasts and participates in wound contraction during tissue repair. At the beginning of the wound repair stage, myofibroblasts pull the wound together to accelerate closure, then undergo apoptosis or return to an inactive state during the remodelling stage [43]. As shown in Figure 8a and Figure S10a, more positive α-SMA expression (blue triangles) of the granulation tissue area was observed in the 5SF-20G group than the other groups on day 7, indicating that the wound preceded the accelerated closure stage. In the intermediate stage of wound closure, the high expression of myofibroblasts is usually related to the duration of active contraction and promotes wound healing further [8]. After 14 days, α-SMA in the blank group retained high expression, while that in the hydrogel groups decreased, suggesting that the wound of the blank group was still in the proliferation phase of repair, but the wounds of the hydrogel groups were undergoing the tissue remodelling stage accompanied by myofibroblast apoptosis (Figure 8b).

Cytokeratin is a pivotal marker for evaluating epidermal differentiation and wound healing to protect epithelial cells from damage [44]. After 7 days, cytokeratin expression (red triangles) was observed in the blank group; however, the superficial layers did not express CK10 (orange dotted frames, Figure 8c), and the cytokeratin in the wound was sunken compared with normal tissue, which may develop into scar tissue in the future (Figure S10b). The hydrogel groups presented an almost negative expression. Expression of CK10 in the blank group was low, with incomplete and disordered keratinocytes on day 14. In the hydrogel groups, CK10 expression was high on the surface of the skin, indicating the formation of complete keratinocyte structures. The epidermal layer of the tissue (5SF-20G) became thinner and gradually approached normal skin tissue (Figure 8d), in addition, the mature hair follicle stained by CK10 was also observed. 

Blood vessels can provide nutrients and oxygen for cells and maintain the growth of newly formed granulation tissue during wound healing [45]. Early regeneration of angiogenesis is critical to the reconstruction of ECM, which is beneficial for wound healing. To assess the effect of the hydrogel groups on the function of blood vessels, CD31 was used to evaluate angiogenesis (red circles) in the wound tissue. As shown in Figure 8e and Figure S10c, well-formed vessels at a higher density were observed in the hydrogel groups (5SF-20G) on day 7, while the vessels in the blank group were anomalistic on days 7 and 14. The number of vessels in the 5SF-20G groups were higher than that of other groups on day 7 and then showed a marked regression on day 14 (Figure 8f). The 5SF-20G hydrogels supported extensive and rapid vascular reformation during the proliferation stage. The regression of blood vessels indicated the wound was in the remodelling phase [46]. These results of IHC evaluation demonstrated that 5SF-20G hydrogels facilitated regeneration at various stages, including re-epithelialisation and revascularisation, to accelerate the efficiency of wound healing.

## 3. Conclusions

In summary, we adopted an effective strategy to construct a physiologically responsive wound dressing using natural SF and Hb protein via moderate mixing and in situ injection. Hydrogels with a 3D porous environment, presented mechanical tunability and adjustable gelation behaviour through Hb-mediated crosslinking. Benefiting by introducing the SF/Hb protein, the induced system and hydrogels demonstrated high biocompatibility, providing adequate cell attachment and proliferation. Furthermore, due to the protection of SF, the enzyme-loaded hydrogels also exhibited high biocatalytic activity and promising antioxidative activity to realise continuous ROS regulation. The 5SF-20G hydrogels as the superior candidate possess rapid gelation time (~5 min), great injectability, high compression modulus (6.4 kPa) and adhesion strength (12.3 kPa) to provide effective support for wounded tissues, which facilitated wound healing by promoting the development of granulation, collagen deposition, re-epithelialisation, and revascularisation. This biomolecular responsive full-protein hydrogel holds great promise for clinical applications, such as wound healing, tissue regeneration, and haemostatic materials.

## 4. Materials and Methods

### 4.1. Materials

Bombyx mori cocoons were provided by a sericulturist (Xuzhou, China). Na_2_CO_3_ (AR, 99.8%), D (+)-glucose anhydrous, hydrogen peroxide (AR, 30%, 10 M), anhydrous ethanol (≥99.7%), Ti(SO_4_)_2_ (AR, ≥96%), H_2_SO_4_ (AR, 95.0−98.0%), and FeSO_4_·7H_2_O (AR, 99.0–101.0%) were purchased from Sinopharm Chemical Reagent Co., Ltd. (Shanghai, China). LiBr (AR, 99%) was purchased from Energy Chemical (Shanghai, China). Hemoglobin (Hb, Bovine Erythrocytes) and glucose oxidase (GOx, Aspergillus niger, Type VII, ≥100,000 units g^−1^ solid) were obtained from Sigma-Aldrich LLC (Shanghai, China). Pronase E from streptomyces griseus (8.126 u mg^−1^), dialysis tubing (MWCO 3500), 1,1-diphenyl-2-picrylhydrazyl (DPPH) and salicylic acid (AR, 99%) were ordered from Shanghai yuanye Bio-Technology Co., Ltd. (Shanghai, China). Tetramethylbenzidine (TMB, BR, 99%) was purchased from Shanghai Baoman Biotechnology Co., Ltd. (Shanghai, China). Dulbecco’s Modified Eagle Medium (DMEM, high glucose: 4.5 mg mL^−1^, HyClone) was obtained from GE Healthcare Life Sciences (Logan, UT, USA). Fatal bovine serum (FBS) was purchased from Zhejiang Tianhang Biotechnology Co., Ltd. (Huzhou, China). Pen/strep and 0.25% Trypsin-EDTA (Gibco) were purchased from Life Technologies Co., Ltd. (Grand Island, NY, USA). The Cell Counting Kit-8 was purchased from 7Sea biotech (Shanghai, China). Calcein AM/propidium iodide (PI) Double Stain Kit and Reactive Oxygen Species (ROS) Assay Kit (2’,7’-Dichlorofluorescin diacetate, DCFH-DA) were obtained from Shanghai Maokang Biotechnology Co., Ltd. (Shanghai, China). Anti-CD31 polyclonal antibody and anti-smooth muscle actin monoclonal antibody were ordered from American Research Products Inc. (Waltham, MA, USA). Cytokeratin 10 polyclonal antibody were purchased from Elabscience Biotechnology Co., Ltd. (Wuhan, China).

### 4.2. Preparation of SF Solution

Firstly, 10 g of silk cocoon pieces were bathed in 2 L of 0.02 M Na_2_CO_3_ solution for 30 min at 98 °C to remove the sericin coating, then the fibroin fibers were rinsed three times and dried for 24 h to obtain degummed silk fibroin. Subsequently, 2 g of fibroin fibers were completely dissolved in 8 mL of 9.3 M LiBr solution for 4 h at 60 °C, and then dialyzed against distilled water using dialysis tubing with 3500 Da MWCO for 48 h to remove the salt. Next, the solution was centrifuged at 12,000 rpm for 15 min to remove the residual impurities. Finally, the SF aqueous solution was obtained and stored at 4 °C until further use [47].

### 4.3. Preparation of SF Hydrogels

Briefly, 3 mg Hb, 10 µL of 450 mg mL^−1^ glucose and 10 µL of GOx (1.0 u mL^−1^, 1.5 u mL^−1^, 2.0 u mL^−1^) were added to the per mL SF solution (1, 3, 5% *w*/*v*) sequentially to ensure the final GOx concentrations were 10, 15 and 20 mu mL^−1^, the specific concentrations are shown in Table S1. The gelation time was evaluated by the vial inversion test, where the gelation time was specified as the time at which the solution no longer flowed after tilting the vials. If no additional explanation is provided, all hydrogels were fabricated by the SF precursor reacting with initiator for two hours at 37 °C.

### 4.4. Fluorescence Spectroscopy

To assess the fluorescence properties of the SF solution and hydrogel, 1.0 mL of 0.5% w/v SF solution was transferred into a 10 mm pathlength quartz cuvette, and the emission spectrum was recorded using a fluorescence spectrometer (F-7000, Hitachi, Japan) from 300 to 500 nm. To avoid the influence of its intrinsic colour, 0.2 mg mL^−1^ Hb was selected as the final concentration; 1 mL of 0.5% *w*/*v* SF solution containing glucose (1.6 mM), GOx (4 u mL^−1^) and Hb (0.2 mg mL^−1^) was added, allowed to react for 30 min at room temperature, and then the emission spectrum of the hydrogel was recorded. The instrumental settings were as follows: PMT voltage, 700 V; scan speed, 12,000 nm min^−1^; slit width, 2.5 nm; EM sampling interval, 10 nm.

### 4.5. Fourier Transform Attenuated Total Reflection Infrared Spectroscopy (ATR–FTIR)

To confirm the transformation of the secondary structure after enzymatic crosslinking, the freeze-dried pure SF solution and SF hydrogels were scanned from 400 to 4000 cm^−1^ for a total of 64 scans with a resolution of 4 cm^−1^ using a Fourier transform infrared spectrometer (Nicolet iS10, Thermo Scientific, Waltham, MA, USA). The secondary structure was quantified by peak-fitting of the amide I band (1600–1700 cm^−1^) using PeakFit v4.12, and the peak assignments were as follows: 1616–1637 cm^−1^ (β-sheet), 1638–1646 cm^−1^ (random coil), 1647–1655 cm^−1^ (α-helix), and 1663–1696 cm^−1^ (β-turn) [41].

### 4.6. Scanning Electron Microscopy (SEM)

The SF hydrogels were frozen with liquid nitrogen, after freeze-drying, the samples were sputtered with a layer of gold at a current intensity of 15 mA for 2 min, and the structure was observed by using a field emission scanning electron microscopy (S-4800, Hitachi, Japan) at a voltage of 5 kV.

### 4.7. Rheological Measurements

To determine the gelation time and mechanical stability, the storage modulus (G′) and loss modulus (G″) were measured using a Thermo-Haake rheometer (RS6000, Thermo Scientific, Waltham, MA, USA) at a frequency of 1 Hz and a shear stress of 1 Pa. Dynamic time sweeps were executed using parallel-plate geometry (diameter: 20 mm, 0.3 mm gap) for 7200 s, after which the dynamic frequency sweeps were implemented on the hydrogels from 10 to 0.1 Hz.

### 4.8. Mechanical and Lap-Shear Testing

SF hydrogels (diameter: 14 mm, height: 5 mm) were fabricated in a Teflon mould, and compression stress–strain curves were collected using an electronic universal testing machine (UTM 2502, Shenzhen, China) with 95% strain at a speed of 5 mm min^−1^. The compression modulus was obtained by calculating the slope of the 5–15% strain. Sequential cyclic strain testing was conducted from 20 to 40% and then to 60% strain at a speed of 20 mm min^−1^. Fresh porcine skin was obtained from markets. The porcine skin was soaked in PBS (pH = 7.4) to remove surface grease. The lap-shear testing was performed on the universal testing machine (68SC-2, Instron, Norwood, MA, USA) to evaluate the shear adhesion strength of the SF hydrogels on porcine skin at a speed of 20 mm min^−1^. The hydrogels were applied to the porcine skin with a bonding area of 25 mm × 20 mm. The shear adhesion strength was calculated by Equation (1):

(1) $$\mathrm{Shear}\;\mathrm{adhesion}\;\mathrm{strength}=\frac{F_{\max}}{S}$$

where F_max_ is the maximum force during shearing and S is the bonding square.

### 4.9. The Degree of Hb Oxidation

Due to the strong absorbance at 405 nm of Hb, the concentrations of glucose, GOx, and Hb were diluted six-fold for final use. Hb oxidation with and without the addition of SF was detected using a UV–vis spectrophotometer (UV-2700, Shimadzu, Kyoto, Japan) from 200 to 700 nm at 25 °C.

### 4.10. Electron Paramagnetic Resonance (EPR) Spectroscopy

EPR spectra were recorded using an electron paramagnetic resonance spectrometer (A300, Bruker, Karlsruhe, Germany). The final concentration of glucose, GOx and Hb were 4.5 mg mL^−1^, 1 u mL^−1^ and 30 mg mL^−1^, respectively. Besides, H_2_O_2_ (1 mM) and Hb (30 mg mL^−1^) was used as a control group to verify the type of protein free radicals. The instrumental settings used to record the 2D spectrum set were: temperature: 77 K, microwave frequency νMW = 9.853 GHz; microwave power PMW = 10.62 mW; modulation amplitude AM = 1.00 G; modulation frequency νM = 100 kHz; sweep width ΔH = 100 G; sweep time ST = 30.72 s; time constant τ = 10.24 ms; conversion time 30.00 ms.

### 4.11. Catalytic Activity of Cascade Enzyme

The catalytic activity of the immobilized and free enzyme was determined based on the oxidation of TMB, the absorbance of the oxidized TMB (ox-TMB) was monitored at 652 nm by a UV-vis spectrophotometer. Firstly, 1 mL of hydrogel (5% SF, glucose: 1.6 mM, GOx: 0.2 mg, and Hb: 20 mg) were fabricated for two hours at 37 °C to ensure the complete consumption of glucose. Secondly, 1 mL of TMB (10 mM, H_2_O), 50 mg of hydrogels and glucose (different concentration) were added into deionised water (final volume 2 mL), the final concentrations of GOx and Hb in abovementioned solution were 5 µg mL^−1^ and 0.5 mg mL^−1^, respectively. In addition, the same concentrations of the free enzyme were used as the control group to detect catalytic activity. Then, the reaction mixture was vigorously vortexed for 30 s, the absorbance at 652 nm was collected continuously at 25 °C for 60 s. The concentrations of the product were calculated according to the Beer–Lambert law (Equation (2)). The Michaelis–Menten kinetic curves (Equation (3)) of enzymes were obtained by plotting the initial reaction velocity. Then the maximal velocity (*V*_max_), Michaelis–Menten constant (*K*_m_), and turnover frequency (*k*_cat_) of the catalytic reaction of the mimic enzyme were calculated by Lineweaver–Burk equation (Equations (4) and (5)).

(2) A=kb[P] 

(3) $$V_{0}=\frac{V_{\max}\left[S\right]}{K_{m}+[S]}$$

(4) $$\frac{1}{V_{0}}=\frac{K_{m}}{V_{\max}}\cdot \frac{1}{\left[S\right]}+\frac{1}{V_{\max}}$$

(5) $$k_{\mathrm{cat}}=\frac{V_{\max}}{\left[E\right]}$$

where *A* represents the absorbance of ox-TMB, *k* is the molar extinction coefficients (35,800 M^−1^ cm^−1^), b is the path length of the sample (1 cm), and [P], [S], and [E] is the concentration of the product, substrate, and enzyme, respectively.

### 4.12. Hydrogels In Vitro Free Radical Scavenging

The antioxidant ability of the SF hydrogel was evaluated using the scavenging capacity of H_2_O_2_, DPPH free radical, and hydroxyl radical (·OH). In brief, 1 mL of SF hydrogel was mashed and then incubated with 3 mL of H_2_O_2_ (1 mM) for 60 min at 37 °C. SF hydrogel incubated with 3 mL of deionised water was regarded as the background group. Then, 1.5 mL of the supernatant was mixed with a 0.5 mL of Ti(SO_4_)_2_ solution (80 mM, 10 mL of H_2_SO_4_ in 40 mL of deionised water) for 5 min, and the concentration of H_2_O_2_ was determined by measuring the absorbance at 410 nm based on the blank control group (3 mL of H_2_O_2_). One millilitre of SF hydrogel was mashed and then incubated with 3 mL of DPPH solution (0.1 mM, anhydrous ethanol) for 60 min at 37 °C in the dark. In addition, SF hydrogels incubated with 3 mL of anhydrous ethanol were regarded as the background group. The absorbance at 517 nm was then determined to calculate the scavenging efficiency based on the blank control group (0.1 mM of DPPH in anhydrous ethanol). Salicylic acid colorimetry was used to detect·OH through the Fenton reaction. One millilitre of SF hydrogel was mashed and then mixed with the following substances sequentially and incubated for 60 min at 37 °C in the dark: 0.15 mL of salicylic acid solution (10 mM, anhydrous ethanol), 0.15 mL of FeSO_4_ solution (10 mM, deionized water), 1.2 mL of deionized H_2_O, and 1.5 mL of H_2_O_2_ (100 mM). The final volume was 3.0 mL. In addition, 0.15 mL of FeSO_4_ solution was replaced with deionised water (using 0.15 mL of deionized water as the background group). The concentration of ·OH was determined by measuring the absorbance at 510 nm based on the blank control group (without SF hydrogel). The scavenging rate obeyed Equation (6):

(6) $$\mathrm{Scavenging}\;\mathrm{Rate}=\left(1-\frac{A_{i}{-A}_{j}}{A_{0}}\right)\;\times \;100\%$$

where A_i_, A_j_, and A_0_ represent the absorbance of the sample, background, and blank control groups, respectively.

### 4.13. Evaluation of Swelling Ratio and In Vitro Enzymatic Degradation

The swelling ratio of wet hydrogels was measured by mass weighing to evaluate the potential of the hydrogel as a wound dressing. First, 200 μL of SF hydrogels were prepared in a centrifuge tube for 2 h at 37 °C, immersed in deionised water at 37 °C, and then weighed at a specific time after removing the outer water using filter paper. This weight was recorded as W_1_ and the initial weight of the wet hydrogel was recorded as W_0_. The swelling ratio was calculated using Equation (7):

(7) $$\mathrm{Swelling}\;\mathrm{Ratio}=\frac{W_{1}{-W}_{0}}{W_{0}}\times \;100\%$$

For the degradation test, 300 μL 0.001 u mL^−1^ of pronase E was added to 200 μL of SF hydrogels and then incubated for 1, 3, 5, and 7 days. SF hydrogels incubated with deionised water were used as the control group. The pronase E solution was changed every 2 days. SF hydrogels were rinsed twice with distilled water, lyophilised, and weighed. The dry weight was recorded as W_t_ and the control weight was recorded as W_0_. The remaining mass obeyed Equation (8):

(8) $$\mathrm{Remaining}\;\mathrm{Mass}=\frac{W_{t}}{W_{0}}\;\times \;100\%$$

### 4.14. Cell Survival and Proliferations

The NIH 3T3 cell lines were obtained from the Cell Bank of Typical Culture Preservation Committee of the Chinese Academy of Sciences. NIH 3T3 cells were cultured at 37 °C and 5% CO_2_ in DMEM supplemented with 1% *v*/*v* penicillin/streptomycin and 10% *v*/*v* FBS. Cytotoxicity was measured using the CCK-8 assay. Briefly, 6 × 10^3^ cells per well were seeded into 96-well plates and incubated for 6 h to induce adherence, then the medium was changed, GOx (2.5, 10, 15, 20 mu mL^−1^) were added to four rows of wells, respectively. GOx (2.5, 10, 15, 20 mu mL^−1^, respectively) and Hb (3 mg mL^−1^) were added to four additional rows of wells, In addition, GOx (2.5, 10, 15, 20 mu mL^−1^, respectively), Hb (3 mg mL^−1^) and SF (1 mg mL^−1^) was added to four additional rows of wells as experimental group. The low concentration of SF (1 mg mL^−1^) was chosen to prevent gel formation at high concentrations. And the glucose existing in the DMEM induced a reaction. These monomers were co-cultivated with cells for 24 and 48 h. The absorbance was measured at 450 nm using a microplate reader (ELx 808 IU, BioTek, Winooski, VT, USA) to calculate the relative survival rates. For surface proliferation, 100 µL of 1% *v*/*w* SF hydrogels were incubated in 48-well plates for 30 min at 37 °C, and then 400 µL of medium containing 1 × 10^4^ cells were seeded on the surface of the hydrogels and incubated for 2 and 7 days. For in situ proliferation, 5 × 10^4^ cells were added to the 100 µL of precursor and reacted for 10 min at 37 °C. Then, 400 µL of medium was added to each well for 2 and 7 days. The medium was replaced with fresh medium once daily. The in vitro proliferation of cells was assessed by live/dead staining (AM: 4 µM, PI: 4.5 µM). Live (green fluorescence) and dead (red fluorescence) cells were observed using an automated digital microscope (LionheartTM FX, BioTek, Winooski, VT, USA) and cell viability was calculated by counting with Image J.

### 4.15. Intracellular ROS Scavenging Ability of SF Hydrogels

In brief, 5 × 10^4^ cells were seeded in a 48-well plate for 12 h and then incubated with the following: (1) 10 µL of PBS as a control; (2) 10 µL of H_2_O_2_ (final concentration 100 µM); (3) 10 µL of H_2_O_2_ and 100 µL of 5SF-10G hydrogel; (4) 10 µL of H_2_O_2_ and 100 µL of 5SF-20G hydrogel. The cells were incubated with DCFH-DA (5 µM in DMEM, without FBS) for 20 min following a 2-h incubation with the additives. The cells were then gently rinsed with PBS three times. Finally, the intracellular ROS level was evaluated by detecting green fluorescence using an inverted fluorescence microscope (Eclipse Ti-S, Nikon, Tokyo, Japan) [39].

### 4.16. Injectability Test

The porcine skin tissue was obtained from markets. A round full-thickness cutaneous wound (10 × 10 mm) area was created on the porcine skin. The precursor (5SF-20G) was loaded into the syringe for 5 min at 37 °C. Then, the SF hydrogels were injected into the wound to verify injectability.

### 4.17. In Vivo Wound Healing

All animals were raised in the animal room at Tongji University, and all procedures involving animals were approved by the Institutional Animal Care and Use Committee (IACUC) of Tongji University (TJAC00321201). A rat model with a full-thickness skin defect was established to evaluate the effect of the SF hydrogel on wound healing. In brief, 30 male Sprague–Dawley (SD) rats (7–8 weeks old) were randomly divided into five groups (*n* = 6). A round full-thickness cutaneous wound (8 × 8 mm) area was created on the back after shaving, 500 μL of SF precursor was injected into the wounds, and the wounds were wrapped with sterile gauze after in situ gelation. The wound area of the rats was photographed daily. On days 7 and 14, three rats per group were sacrificed, and the wounded tissue was fixed in 10% (*v*/*v*) buffered formaldehyde, dehydrated with a graded ethanol series, and embedded in paraffin. The specimen was sliced into 4-μm-thickness sections and analysed by haematoxylin and eosin (H&E) staining and Masson’s trichrome staining to evaluate the degree of inflammation and collagen deposition. The cellular and molecular mechanisms underlying wound repair was analysed by immunolabel staining for CD 31, CK10, and α-SMA according to standard protocols. Expression levels of α-SMA, CK10, and CD31 were quantified by Image J (IHC Toolbox). The wound area was measured using Image J, and the wound healing rate was calculated using Equation (9):

(9) $$\mathrm{Wound}\;\mathrm{Healing}\;\mathrm{Rate}=\frac{W_{0}{-W}_{1}}{W_{0}}\times 100\%$$

where W_0_ represents the initial wound area and W_1_ represents the initial wound area at each time point.

### 4.18. Statistical Analyses

The unpaired Student’s *t*-test was used to compare differences between two separate groups (* *p* < 0.05, ** *p* < 0.01, *** *p* < 0.001). All data are expressed as mean ± standard deviation for *n* ≥ 3.

## Data Availability

Data is contained within the article.

## Supplementary Materials

The following are available online at https://www.mdpi.com/article/10.3390/gels8010056/s1. Table S1. Concentrations of each ingredient in SF hydrogels; Figure S1. Gelation times of varying SF and GOx concentration at 37 °C; Figure S2. Mechanism of SF hydrogel formation; Figure S3. Characterization of hydrogel morphology; Figure S4. Rheological properties of SF hydrogels under different enzymatic systems; Figure S5. UV−vis spectra of (a) H_2_O_2_, (b) DPPH and (c) ·OH after being scavenged by the SF hydrogels for one hour; Figure S6. H_2_O_2_, DPPH and ·OH scavenging rate of the SF hydrogels (5SF-10G, 5SF-20G); Figure S7. Mass Remaining of silk hydrogels after enzymatic degradation; Figure S8. The oxidative stress in cells incubated with SF hydrogels (5SF-10G and 5SF-20G) was monitored via a ROS probe (DCFH-DA); Figure S9. Wound healing abilities of SF hydrogels; Figure S10. Wound healing abilities of SF hydrogels; Movies S1 and S2. The demonstration of injectability and formability of SF hydrogels.
