# Supplementary material for: GOx/Hb Cascade Oxidized Crosslinking of Silk Fibroin for Tissue-Responsive Wound Repair
Source: Gels. 2022 Jan 12;8(1):56. doi: 10.3390/gels8010056 (PMC8774987; doi:10.3390/gels8010056)
Supplement: Supplementary file 1 [file gels-08-00056-s001.zip › gels-1526282-supplementary.pdf]

Supplementary Materials

GOx/Hb Cascade Oxidized Crosslinking of Silk Fibroin for Tissue-Responsive Wound Repair

Hongdou Shen [1,2,†], Pei Wang [1,†], Xiaoke Han [2], Mengli Ma [2], Yinghui Shang [2], Ye Ju [2], Saiji Shen [2], Feng Yin [1,*] and Qigang Wang [2,*]

[1] Department of Joint Surgery, Shanghai East Hospital, School of Medicine, Tongji University, Shanghai 200120, P.R. China; shenhongdou2018@163.com (H.S.); drwp007@163.com (P.W.)
[2] School of Chemical Science and Engineering, Tongji University, Shanghai 200092, P.R. China; 1931002@tongji.edu.cn (X.H.); 17334567254@163.com (M.M.); shangdiyinghui@163.com (Y.S.); juye1997@hotmail.com (Y.J.); shensaiji@tongji.edu.cn (S.S.)
* Correspondence: 1300229@tongji.edu.cn (F.Y.); wangqg66@tongji.edu.cn (Q.W.)
† These authors contributed equally to this work.

Results and Discussion

Table S1. Concentrations of each ingredient in SF hydrogels.

Sample	SF	Glucose	GOx	Hb
1SF-10G			10 mu mL^{-1}	
1SF-15G	1.0% w/v		15 mu mL^{-1}	
1SF-20G			20 mu mL^{-1}	
3SF-10G			10 mu mL^{-1}	
3SF-15G	3.0% w/v	4.5 mg mL^{-1}	15 mu mL^{-1}	3 mg mL^{-1}
3SF-20G			20 mu mL^{-1}	
5SF-10G			10 mu mL^{-1}	
5SF-15G	5.0% w/v		15 mu mL^{-1}	
5SF-20G			20 mu mL^{-1}	

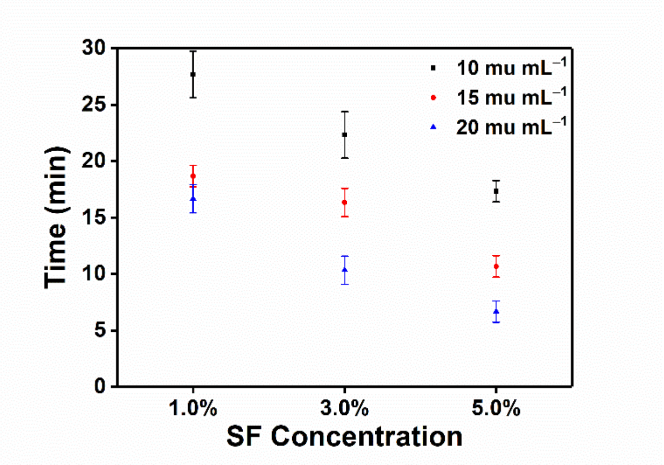

Figure S1. Gelation times of varying SF and GOx concentration at 37 °C. Error bars represent the mean ± standard deviation (s.d.); n = 3.

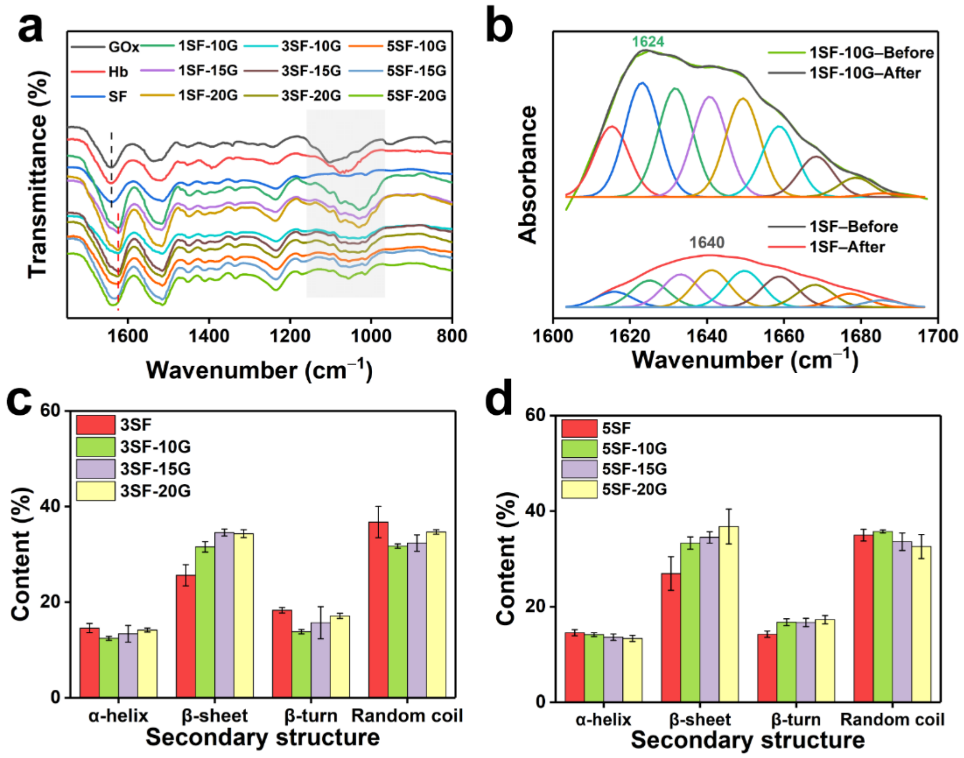

Figure S2. Mechanism of SF hydrogel formation. (a) FTIR spectra of GOx, Hb, SF hydrogels. (b) Peak fitting diagram of 1SF, 1SF-10G were performed using a PeakFit v4.12. (c, d) The results of second structure of SF hydrogels by peak fitting, Error bar represent mean ± s.d.; n ≥ 3.

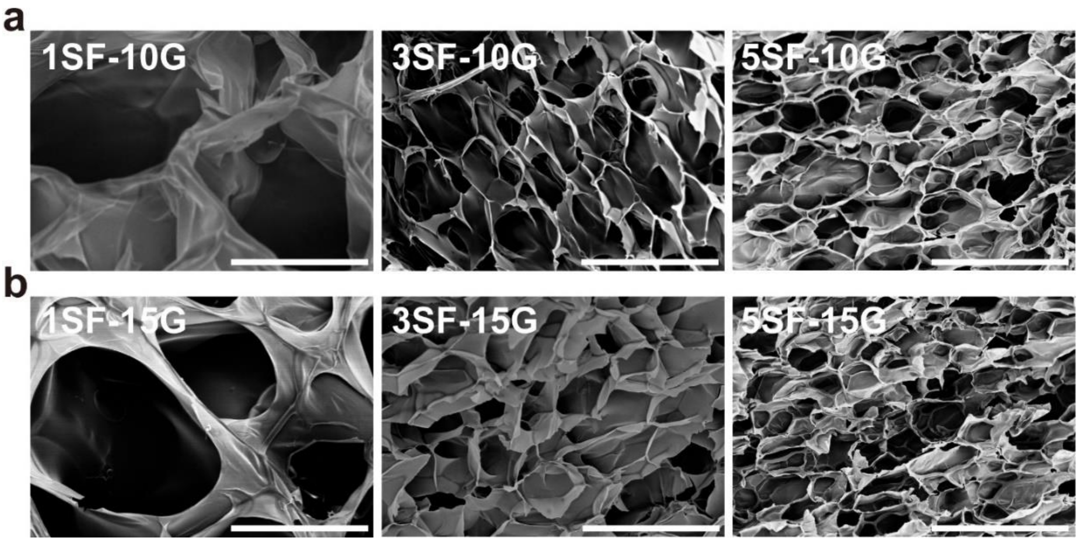

Figure S3. Characterization of hydrogel morphology. The 3D porous structure of SF hydrogel (a) SF/glucose/GOx (10 mu mL^{-1})/Hb, (b) SF/glucose/GOx (15 mu mL^{-1})/Hb with different SF concentration. Scale bars are 50 μm.

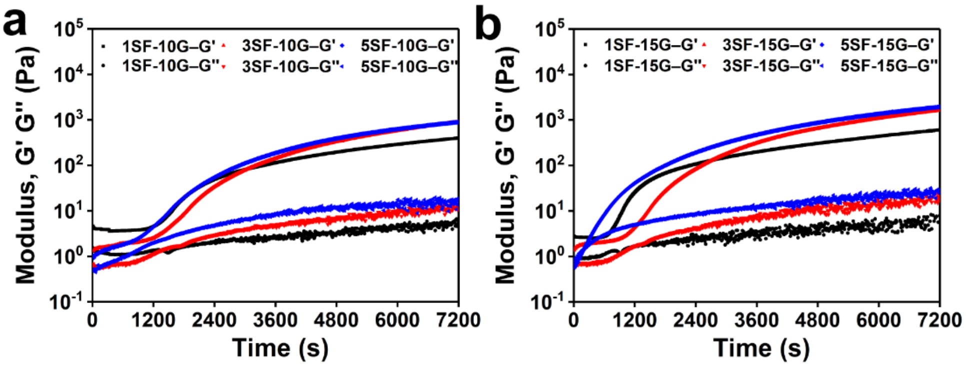

Figure S4. Rheological properties of SF hydrogels under different enzymatic systems. The storage modulus (G′) and loss modulus (G″) of (a) SF/glucose/GOx (10mu mL^{-1})/Hb and (b) SF/glucose/GOx (15mu mL^{-1})/Hb hydrogels.

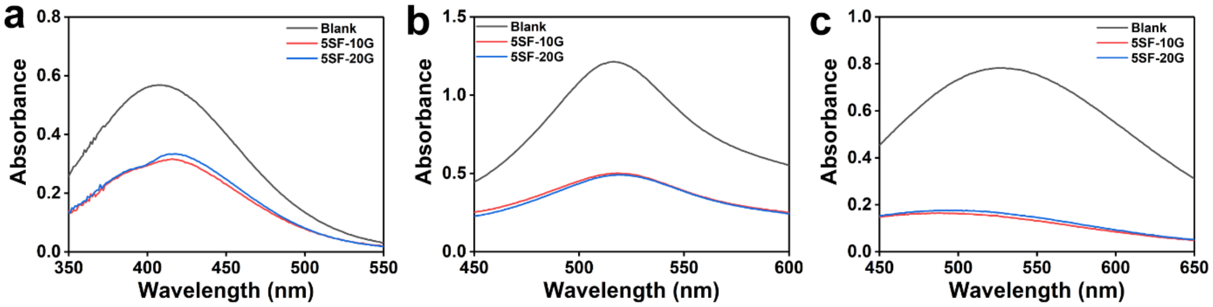

Figure S5. UV−vis spectra of (a) H_2O_2, (b) DPPH and (c) ·OH after being scavenged by the SF hydrogels for one hour.

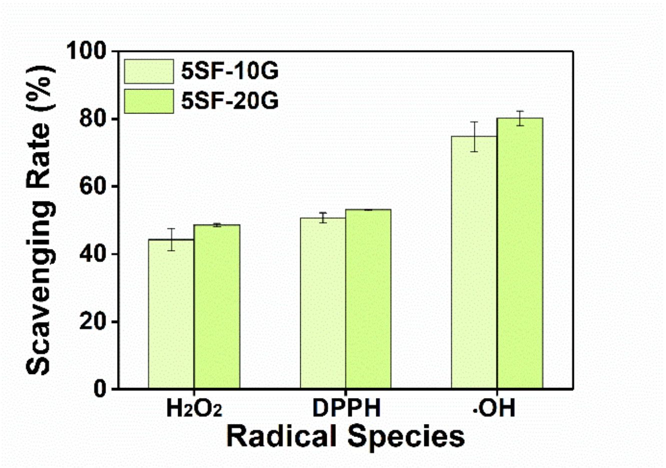

Figure S6. H_2O_2, DPPH and ·OH scavenging rate of the SF hydrogels (5SF-10G, 5SF-20G). Error bar represent mean ± s.d.; n ≥ 3.

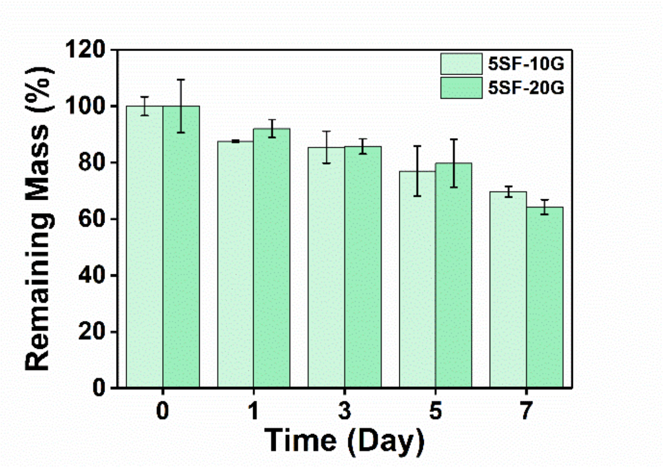

Figure S7. Mass Remaining of silk hydrogels after enzymatic degradation. Error bar represent mean ± s.d.; n ≥ 3.

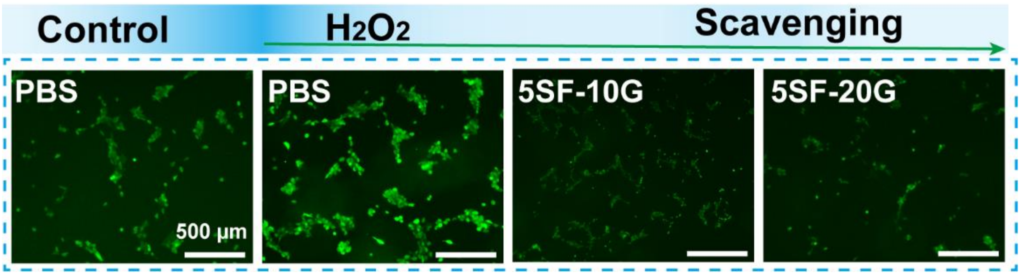

Figure S8. The oxidative stress in cells incubated with SF hydrogels (5SF-10G and 5SF-20G) was monitored via a ROS probe (DCFH-DA). Scale bar are 500 µm.

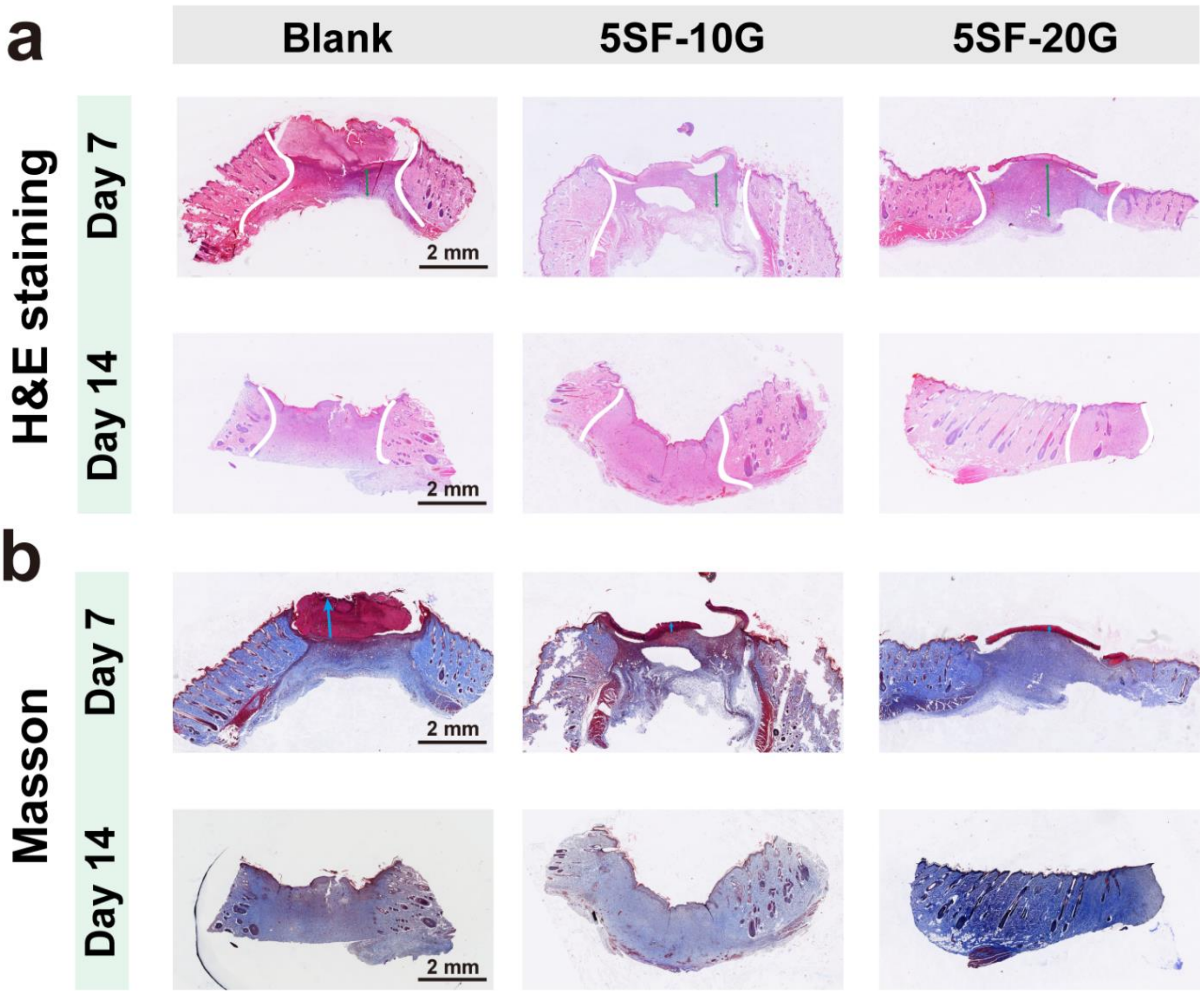

Figure S9. Wound healing abilities of SF hydrogels. (a) H&E staining of the wound tissues on Days 7 and 14. White solid lines and green double-headed arrows represent the range and thickness of granulation tissue (b) Masson's trichrome staining of the wound tissues on Days 7 and 14. Blue arrows represent the residual scab, Scale bar are 2 mm.

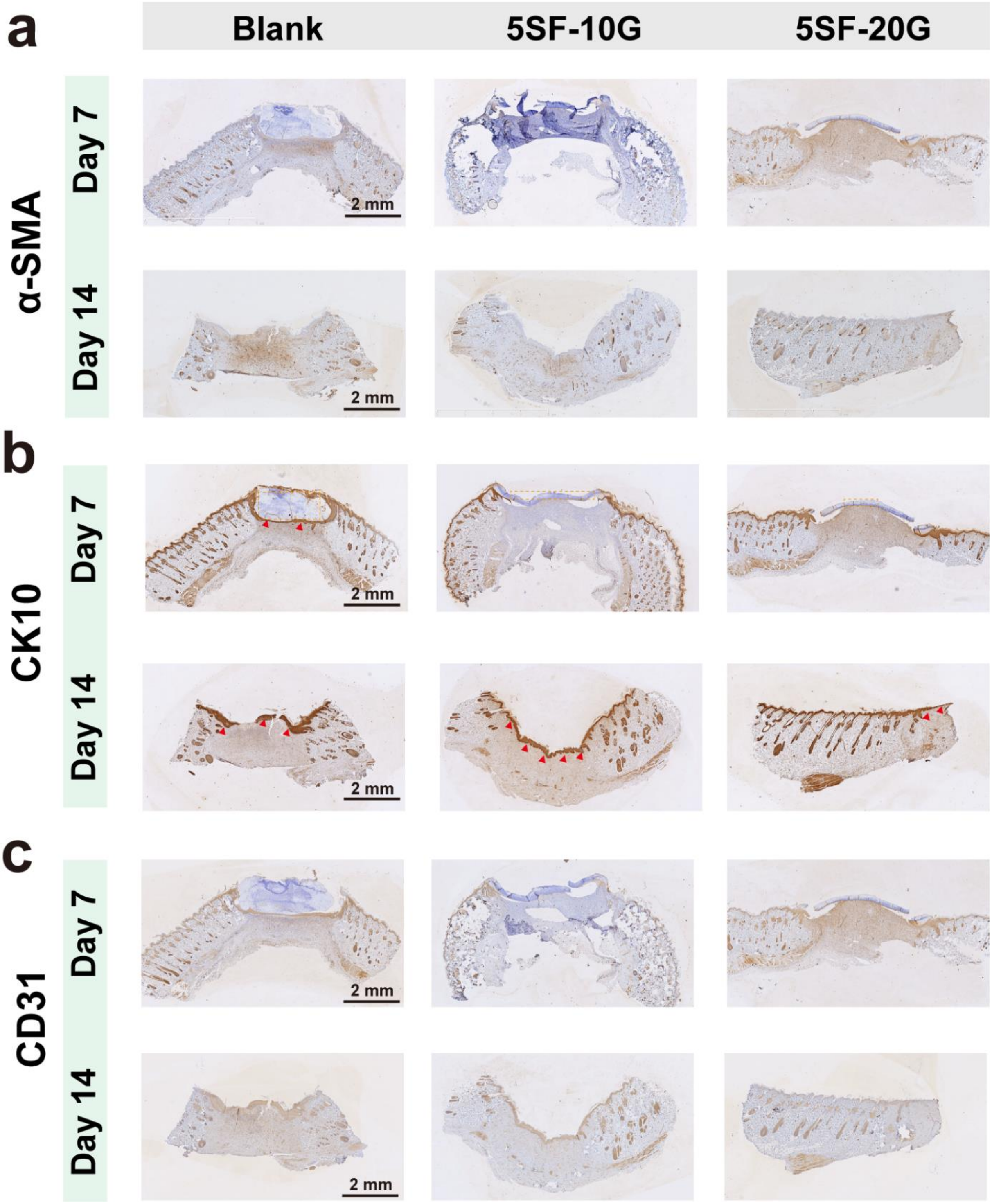

Figure S10. Wound healing abilities of SF hydrogels. (a) α-SMA staining, showing myofibroblasts on days 7 and 14. (b) CK10 staining, showing keratin in skin on days 7 and 14, orange dotted frames and red triangles indicated the negative and positive expression of keratin, respectively. (c) CD31 staining images, representing the extent of vascularization on days 7 and 14. Scale bars are 2 mm.
